# Molecular Targets for Novel Therapeutics in Pediatric Fusion-Positive Non-CNS Solid Tumors

**DOI:** 10.3389/fphar.2021.747895

**Published:** 2022-01-20

**Authors:** Wen-I Chang, Claire Lin, Nicholas Liguori, Joshua N. Honeyman, Bradley DeNardo, Wafik El-Deiry

**Affiliations:** ^1^ Laboratory of Translational Oncology and Experimental Cancer Therapeutics, The Warren Alpert Medical School, Brown University, Providence, RI, United States; ^2^ Pediatric Hematology/Oncology, The Warren Alpert Medical School, Brown University, Providence, RI, United States; ^3^ The Joint Program in Cancer Biology, Brown University and Lifespan Health System, Providence, RI, United States; ^4^ Pediatric Surgery, The Warren Alpert Medical School, Brown University, Providence, RI, United States; ^5^ Department of Pathology and Laboratory Medicine, The Warren Alpert Medical School, Brown University, Providence, RI, United States; ^6^ Cancer Center at Brown University, The Warren Alpert Medical School, Brown University, Providence, RI, United States; ^7^ Hematology/Oncology Division, Department of Medicine, Lifespan Health System and Brown University, Providence, RI, United States

**Keywords:** sarcoma, pediatric, fusion-positive, molecular targets, solid tumors

## Abstract

Chromosomal fusions encoding novel molecular drivers have been identified in several solid tumors, and in recent years the identification of such pathogenetic events in tumor specimens has become clinically actionable. Pediatric sarcomas and other rare tumors that occur in children as well as adults are a group of heterogeneous tumors often with driver gene fusions for which some therapeutics have already been developed and approved, and others where there is opportunity for progress and innovation to impact on patient outcomes. We review the chromosomal rearrangements that represent oncogenic events in pediatric solid tumors outside of the central nervous system (CNS), such as Ewing Sarcoma, Rhabdomyosarcoma, Fibrolamellar Hepatocellular Carcinoma, and Renal Cell Carcinoma, among others. Various therapeutics such as CDK4/6, FGFR, ALK, VEGF, EGFR, PDGFR, NTRK, PARP, mTOR, BRAF, IGF1R, HDAC inhibitors are being explored among other novel therapeutic strategies such as ONC201/TIC10.

## Introduction

Pediatric cancer rates have been rising the past few decades, and approximately 400,000 new cases are diagnosed globally each year (Ward et al., 2019a). While the overall 5-year survival rate for pediatric oncology patients in the United States is 84%, outcomes are generally worse in pediatric solid tumors outside of the central nervous system (CNS), especially in cases of up-front metastases, recurrence, or progressive disease (Siegel et al., 2020). Pediatric sarcomas account for approximately 10% of all childhood tumors, and include both soft tissue and bone-related tumors. Pediatric carcinomas, including liver tumors such as fibrolamellar hepatocellular carcinoma, are less common. Of all pediatric sarcoma patients, recurrence occurs in 30–40% of cases, with patients facing poor event-free survival odds after relapse (Stahl et al., 2011; Duchman et al., 2015; Smeland et al., 2019). Similarly, pediatric carcinoma patients who present with up-front metastasis can have an overall survival rate of less than 20% (Schmid and von Schweinitz, 2017).

A subset of extra-cranial pediatric solid tumors are fusion-positive tumors, which are characterized by abnormal chromosomal rearrangements (e.g., translocations, insertions, inversions, and deletions) that result in the fusion of two disparate genes. Fusion genes can lead to constitutively activated oncogenes when a proto-oncogene is upregulated by the promoter and activator sequences of the partner gene in the fusion (Dupain et al., 2017). Additionally, the encoded chimeric oncoprotein can act as an aberrant transcription factor, driving downstream activation of pathways involved in tumorigenesis. Gene fusions may also result in deletion of tumor-suppressor genes, allowing for cancer cell transformation (Dupain et al., 2017).

In the past few decades, detection of these fusions has been of great interest in both basic cancer research and in the clinical setting, as fusions can be used as diagnostic markers, prognostic indicators, and therapeutic targets in the treatment of disease (Table 1). Clinical utility with screening fusion panels in pediatric solid tumors and hematological malignancies has been previously demonstrated, highlighting the need for targeted therapies in fusion-positive pediatric cancers (Chang et al., 2019).

**TABLE 1:** Different gene fusions and their corresponding tumor and current significance(s).

Gene fusion	Tumor	Significance
*EWS-FLI1*	Ewing sarcoma	Prognostic de Alava et al. (2000)
		Direct therapeutic target García-Domínguez et al. (2018), Spriano et al. (2019)
*PAX-FOXO1*	Alveolar rhabdomyosarcoma	Diagnostic Wexler and Ladanyi (2010)
		Prognostic Arnold and Barr (2017)
*ETV6-NRTK3*	Infantile fibrosarcoma	Diagnostic Bourgeois et al. (2000)
		Direct therapeutic target Dunn (2020)
*TFE3-ASPSCR1*	Alveolar soft part sarcoma	Diagnostic Aulmann et al. (2007)
Fusion partner-*ALK*	Inflammatory myofibroblastic tumor	Diagnostic Cook et al. (2001)
		Direct therapeutic target Trahair et al. (2019), Fordham et al. (2020)
*DNAJB1-PRKACA*	Fibrolamellar hepatocellular carcinoma	Diagnostic Honeyman et al. (2014)
Fusion partner-*TFE3*	Renal cell carcinoma	Diagnostic Akgul et al. (2021)
*SS18-SSX*	Synovial sarcoma	Diagnostic Clark et al. (1994), Shipley et al. (1994)
*EWS-WT1*	Desmoplastic small round cell tumors (DSRCT)	Diagnostic Ladanyi and Gerald (1994)
*EWS-ATF1*	Clear cell sarcoma	Diagnostic Antonescu et al. (2002)

Current therapeutic strategies focus on decreasing long-term treatment toxicity while maintaining excellent outcomes for low-risk patients, while improving outcomes for high-risk patients, often through treatment intensification. In pediatric solid tumors, the combination of cytotoxic chemotherapy, radiation, and surgery has been the standard in pediatric patients for over 2 decades. With the advent of precision medicine, and subsequent increasing understanding of molecular drivers of disease, such as fusion oncogenes, emphasis is currently placed on investigating targeted treatment options.

## Ewing Sarcoma

Ewing Sarcoma (ES) is the second most common pediatric bone cancer. ES tumors are thought to arise from mesenchymal progenitor cells in bones and soft tissues in both children and adolescents. ES is most frequently driven by the fusion of a FET/TET family of RNA-binding proteins and an ETS family of transcription factors, with approximately 85–90% of ES cases harboring a common *EWSR1-FLI1* fusion (Brohl et al., 2014). These tumors have relatively quiet genome with a low mutational burden, as most of the tumors only have fusion gene rearrangements identified. *STAG2* loss is identified in 15% of ES (Brohl et al., 2014). There is inconsistent information about whether mutations in *STAG2*, *CDKN2A* and *TP53* lead to less favorable prognoses (Honoki et al., 2007; Brohl et al., 2014; Lerman et al., 2015; Brohl et al., 2017).

ES disease progression can be further monitored using a liquid biopsy technique (Hayashi et al., 2016). Liquid biopsies detect circulating tumor DNA (ctDNA) in the peripheral blood. There are promising initial results using capture based next generation sequencing or digital droplet PCR to function as a disease monitoring tool. Circulating tumor DNA has been shown to be effective at detecting disease. One study of ctDNA in the blood found detectable levels in 53% patients tested (Shulman et al., 2018). Detectable ctDNA is associated with inferior outcomes in these patients and can inform how to proceed with management of the disease (Shulman et al., 2018).

Treatment of ES is *via* a three-pronged approach. Surgery, radiation, and chemotherapy are the mainstays of up-front therapy in ES. Surgery to remove the tumor requires a wide resection (Adzhubei et al., 2010). ES has also expressed sensitivity to radiation therapies in attempts to treat and manage the disease (Ewing, 19211972). It is possible to use radiation therapies as definitive local control for inoperable tumors and radiation may also be used pre- or post-operatively to combat tumor progression or in the setting of marginal or intralesional resection (Zöllner et al., 2021). The optimal utilization of radiation therapy is unclear prior to surgery, but the benefit can be significant (Zöllner et al., 2021). This multimodal treatment strategy of combining cytotoxic chemotherapy with radiation and/or surgery has resulted in a 5-year survival rate of 65–75% in patients with localized disease. In addition, patients with metastatic disease suffer from a survival rate of less than 30% with no appreciable improvements over the past 30 years (Gaspar et al., 2015).

The EWSR1-FLI1 oncoprotein acts as an aberrant transcription factor that binds to the canonical ETS binding site and GGAA microsatellite sequences, thereby deregulating cell cycle genes involved in checkpoint control. Genes regulating cell function, cell migration, signal transduction, and chromatin structure are also affected, leading to malignant transformation.

Several studies have shown the oncogenic effect of the *EWSR1-FLI1* fusion, as inhibition of ES cell growth occurs when the EWSR1-FLI1 fusion protein is depleted (Herrero-Martín et al., 2009). This makes for a valuable target for therapies aimed at treating this disease. Despite being recognized as a valuable therapeutic target over 25 years ago, there have been relatively few steps towards inhibiting its tumorigenic characteristics.

Lysine-specific histone demethylase 1 (LSD1) is a part of the nucleosome remodeling and deacetylase (NuRD) co-repressor complex. It is recruited by *EWSR1-FLI1* to regulate transcription. *In vitro* and *in vivo* experiments with LSD1 inhibition have resulted in reversal of the EWSR1-FLI1 oncogenic activity (Sankar et al., 2013; Sankar et al., 2014). Several of the LSD1 inhibitors have begun to enter clinical trials.

The insulin-like growth factor 1 receptor (IGF-1R) pathway is deregulated by the *EWSR1-FLI1* translocation and is an exciting potential target for targeted therapies. There are currently trials ongoing that display at least a partial response in 11.7% of participants, when the IGF-1R inhibitor is administered alone or in combination (van Maldegem et al., 2016). However, a phase II trial combining an IGF-1R inhibitor with an mTOR inhibitor did not show any benefit (Malempati et al., 2012).

The poly (ADP-ribose) polymerase (PARP) pathway is essential for detection of DNA repair and detection of DNA stand breakage in tumor cells. A phase II trial examined 12 patients with ES who were administered 400 mg of oral olaparib, a PARP inhibitor, twice, daily (Choy et al., 2014). While there were no significant toxicities to report, tumor response was not seen in any of the 12 patients, although four of the 12 patients reported stable disease with a median time for progression-free survival (PFS) of 5.7 weeks (Choy et al., 2014). Additionally, there is an open clinical study ongoing testing the PARP inhibitor niraparib to refine dosing and determine dose-limiting toxicities. Similarly, this trial also evaluates escalating doses of temozolomide and/or irinotecan in patients who have recurrent or progressive Ewing Sarcoma that has failed previous treatment (clinicaltrials.gov, NCT 02044120).

There is optimism in a novel ES treatment that focuses on the combination of epigenetic drugs vorinostat and HCI-2509 to inhibit EWSR1-FLI1 and suppress tumor growth. This study analyzed proliferation and cell viability in ES cell lines, showing a synergistic combination of the two epigenetic drugs. These drugs also increased the amount of apoptosis induced in these tumor cells (García-Domínguez et al., 2018). The mechanistic interaction between these epigenetic therapies and EWSR1-FLI1 is not currently well known. However, many of the tumor cells analyzed were stuck in their G1 phase, and apoptosis was induced (García-Domínguez et al., 2018). Treatment with these agents, individually or in combination, resulted in a significant decrease in levels of *EWSR1-FLI1* mRNA and protein (García-Domínguez et al., 2018). This result was also confirmed in patient-derived xenograft mice.

Additionally, preclinical work examining the effect of small molecule inhibitors on ETS-transcription factors is ongoing. The molecule YK-4-279 is a small molecule inhibitor that binds to EWSR1-FLI1 and blocks its interaction with RNA helicase A (RHA) (Spriano et al., 2019). RHA is required for efficient EWSR1-FLI1 activity. When the RHA- EWSR1-FLI1 interaction is blocked, ES cellular growth is inhibited, and tumor proliferation is halted. This interaction results in cell cycle inhibition and promotion of apoptosis, resulting in reduced growth in tumor cells (Erkizan et al., 2009). RHA-EWSR1-FLI1 interactions serve as a unique point of optimism in development of ES targeted therapies. The clinical derivative of YK-4-279, known as TK-216, is currently in Phase I clinical trials for patients with refractory or relapsed ES (clinicaltrials.gov, NCT02657005).

Further investigation into the efficacy of targeted therapies and their impact on ES outcomes is needed. There are currently numerous clinical trials underway that are focusing on molecular targets to effectively treat ES and result in better outcomes. Unlike kinase fusions, the *EWSR1-FLI1* translocation has yet to be successfully targeted with novel therapies. This represents an area of research that needs further investigation and analysis of *in vivo* and *in vitro* studies, as well as clinical trial protocols. Perhaps most intriguing are studies involving small molecule-based therapies. These trials are summarized in Table 2 and are a point of optimism in advancing ES treatment options.

**TABLE 2:** Small Molecule Clinical Trials that are Recruiting or Active for Adolescent or Pediatric Ewing Sarcoma Patients (All Data from ClinicalTrials.gov).

Name of study	Phase	Target	Small molecule treatment	Identifier
9-ING-41 with Chemotherapy in Sarcoma	II	GSK-3β	9-ING-41	NCT05116800
Cabozantinib-S-Malate in treating younger patients with recurrent, refractory, or Newly diagnosed Sarcomas, Wilms Tumor, or Other Rare Tumors	II	AXL, MET, RET, VEGFR2	Cabozantinib-s-malate	NCT02867592
Ensartinib in treating patients with relapsed or Refractory advanced solid Tumors, Non-Hodgkin Lymphoma, or Histiocytic disorders with ALK or ROS1 genomic alterations (A Pediatric MATCH Treatment Trial)	II	ALK	Ensartinib	NCT03213652
Targeted therapy directed by Genetic testing in treating pediatric patients with relapsed or Refractory advanced solid Tumors, Non-Hodgkin Lymphomas, or Histiocytic disorders (The Pediatric MATCH Screening Trial)	II	FGFR, ALK, IDH1, NTRK, PARP, CDK4/6, PI3K/mTOR, RET, MEK, EZH2, HRAS, MAPK, BRAF	Ensartinib, erdafitinib, ivosidenib, larotrectinib, olaparib, palbociclib, samotolisib, selpercatinib, selumetinib, tazemetostat, tipifarnib, ulixertinib, vemurafenib	NCT03155620
Erdafitinib in treating patients with relapsed or Refractory advanced solid Tumors, Non-Hodgkin Lymphoma, or Histiocytic Disorders with FGFR Mutations (A Pediatric MATCH Treatment Trial)	II	FGFR	Erdafitinib	NCT03210714
Ivosidenib in Treating Patients with Advanced Solid Tumors, Lymphoma, or Histiocytic Disorders with IDH1 Mutations (A Pediatric MATCH Treatment Trial)	II	IDH1	Ivosidenib	NCT04195555
Larotrectinib in Treating Patients with Relapsed or Refractory Advanced Solid Tumors, Non-Hodgkin Lymphoma, or Histiocytic disorders with NTRK Fusions (A Pediatric MATCH Treatment Trial)	II	NTRK	Larotrectinib	NCT03213704
Olaparib in treating patients with relapsed or Refractory advanced solid Tumors, Non-Hodgkin Lymphoma, or Histiocytic disorders with defects in DNA damage repair genes (A Pediatric MATCH Treatment Trial)	II	PARP	Olaparib	NCT03233204
Palbociclib in treating patients with relapsed or Refractory Rb positive advanced solid Tumors, Non-Hodgkin Lymphoma, or Histiocytic disorders with activating alterations in cell cycle genes (A Pediatric MATCH Treatment Trial)	II	CDK4/6	Palbociclib	NCT03526250
Palbociclib + Ganitumab in Ewing Sarcoma	II	CDK4/6, IGF1R	Palbociclib, Ganitumab	NCT04129151
SARC024: A Blanket Protocol to study oral Regorafenib in patients with selected sarcoma subtypes	II	VEGFR, PDGFR-β, FGFR, KIT, RET, RAF	Regorafenib	NCT02048371
A Phase II study evaluating efficacy and safety of Regorafenib in patients with Metastatic Bone Sarcomas (REGOBONE)	II	VEGFR, PDGFR-β, FGFR, KIT, RET, RAF	Regorafenib	NCT02389244
Samotolisib in treating patients with Relapsed or Refractory Advanced Solid Tumors, Non-Hodgkin Lymphoma, or Histiocytic disorders with TSC or PI3K/MTOR mutations (A Pediatric MATCH Treatment Trial)	II	PI3K, mTOR	Samotolisib	NCT03213678
Selpercatinib for the treatment of Advanced Solid Tumors, Lymphomas, or Histiocytic disorders with activating RET gene alterations, a Pediatric MATCH Treatment Trial	II	RET	Selpercatinib	NCT04320888
Sirolimus in combination with Metronomic Chemotherapy in children with recurrent and/or refractory solid and CNS Tumors (AflacST1502)	II	mTOR	Sirolimus	NCT02574728
Tazemetostat in treating patients with Relapsed or Refractory Advanced Solid Tumors, Non-Hodgkin Lymphoma, or Histiocytic disorders with EZH2, SMARCB1, or SMARCA4 Gene Mutations (A Pediatric MATCH Treatment Trial)	II	EZH2, SMARCB1, SMARCA4	Tazemetostat	NCT03213665
Tipifarnib for the Treatment of Advanced Solid Tumors, Lymphoma, or Histiocytic Disorders with HRAS Gene Alterations, a Pediatric MATCH Treatment Trial	II	HRAS	Tipifarnib	NCT04284774
Vemurafenib in treating patients with Relapsed or Refractory Advanced Solid Tumors, Non-Hodgkin Lymphoma, or Histiocytic disorders with BRAF V600 Mutations (A Pediatric MATCH Treatment Trial)	II	BRAF	Vemurafenib	NCT03220035
Dasatinib, Ifosfamide, Carboplatin, and Etoposide in treating young patients with Metastatic or Recurrent Malignant Solid Tumors	I/II	BCR/ABL, Src Family Tyrosine kinase (SFK)	Dasatinib	NCT00788125
Study of entrectinib (Rxdx-101) in children and Adolescents with locally Advanced or Metastatic Solid or Primary CNS Tumors and/or Who have no satisfactory treatment options (STARTRK-NG)	I/II	NTRK, ROS1, ALK	Entrectinib	NCT02650401
Study of lenvatinib in combination with Everolimus in recurrent and refractory pediatric solid tumors, including central nervous system tumors	I/II	VEGFR, mTOR	Lenvatinib, everolimus	NCT03245151
Pharmacokinetic Study of PM01183 in Combination with Irinotecan in Patients with Selected Solid Tumors	I/II	DNA minor groove	Lurbinectedin	NCT02611024 (not pediatric)
Study of Onivyde with Talazoparib or Temozolomide in children with Recurrent Solid Tumors and Ewing Sarcoma	I/II	PARP	Talazoparib, Onivyde (irinotecan liposomal)	NCT04901702
Abemaciclib in children with DIPG or Recurrent/Refractory Solid Tumors (AflacST1501)	I	CDK4/6	Abemaciclib	NCT02644460
A study of Abemaciclib in combination with Temozolomide and Irinotecan and Abemaciclib in combination with Temozolomide in children and young adult participants with Solid Tumors	I	CDK4/6	Abemaciclib	NCT04238819
Cabozantinib with Topotecan-Cyclophosphamide	I	AXL, MET, RET, VEGFR2	Cabozantinib	NCT04661852
Dose Escalation study of CLR 131 in children, Adolescents, and young adults with Relapsed or Refractory Malignant Tumors including but not limited to Neuroblastoma, Rhabdomyosarcoma, Ewing Sarcoma, and Osteosarcoma (CLOVER-2)	I	Lipid rafts	CLR 131 (phospholipid drug conjugate)	NCT03478462
Phase I study of Olaparib and Temozolomide for Ewing Sarcoma or Rhabdomyosarcoma	I	PARP	Olaparib	NCT01858168
Study of Palbociclib combined with Chemotherapy in Pediatric patients with Recurrent/Refractory Solid Tumors	I	CDK4/6	Palbociclib	NCT03709680
Pbi-shRNA™ EWS/FLI1 Type 1 LPX in subjects with advanced Ewing’s Sarcoma	I	Type 1 junction EWS-FLI1 translocation	pbi-shRNA™ EWS/FLI1 Type 1 LPX	NCT02736565
A Phase I dose finding study in children with Solid Tumors Recurrent or Refractory to standard therapy	I	VEGFR, PDGFR-β, FGFR, KIT, RET, RAF	Regorafenib	NCT02085148
Clinical Trial of SP-2577 (Seclidemstat) in patients with Relapsed or Refractory Ewing or Ewing-related Sarcomas	I	LSD1	Seclidemstat	NCT03600649
TK216 in patients with Relapsed or Refractory Ewing Sarcoma	I	EWS-FLI1	TK216	NCT02657005
Vorinostat in combination with Chemotherapy in Relapsed/Refractory Solid Tumors and CNS Malignancies (NYMC195)	I	HDAC	Vorinostat	NCT04308330

Abemaciclib is a small molecule inhibitor of CDK4 and CDK6, and it is currently FDA approved for HR+/HER2− advanced breast cancer (Dowless et al., 2018). Additionally, it is available in lung cancer and other solid tumors. In the laboratory, abemaciclib showed tumor suppression in ES cell lines by blocking the G1 phase of the cell cycle (Dowless et al., 2018). Additionally, when cytokine secretion, antigen presentation, and interferon pathway upregulation were measured, abemaciclib was shown to have anti-inflammatory effects (Dowless et al., 2018).

This small molecule is currently being evaluated in two phase I clinical trials in pediatric solid tumors, including ES, either as monotherapy, or in combination with temozolomide and irinotecan, or with temozolomide alone.

**FIGURE 1:**
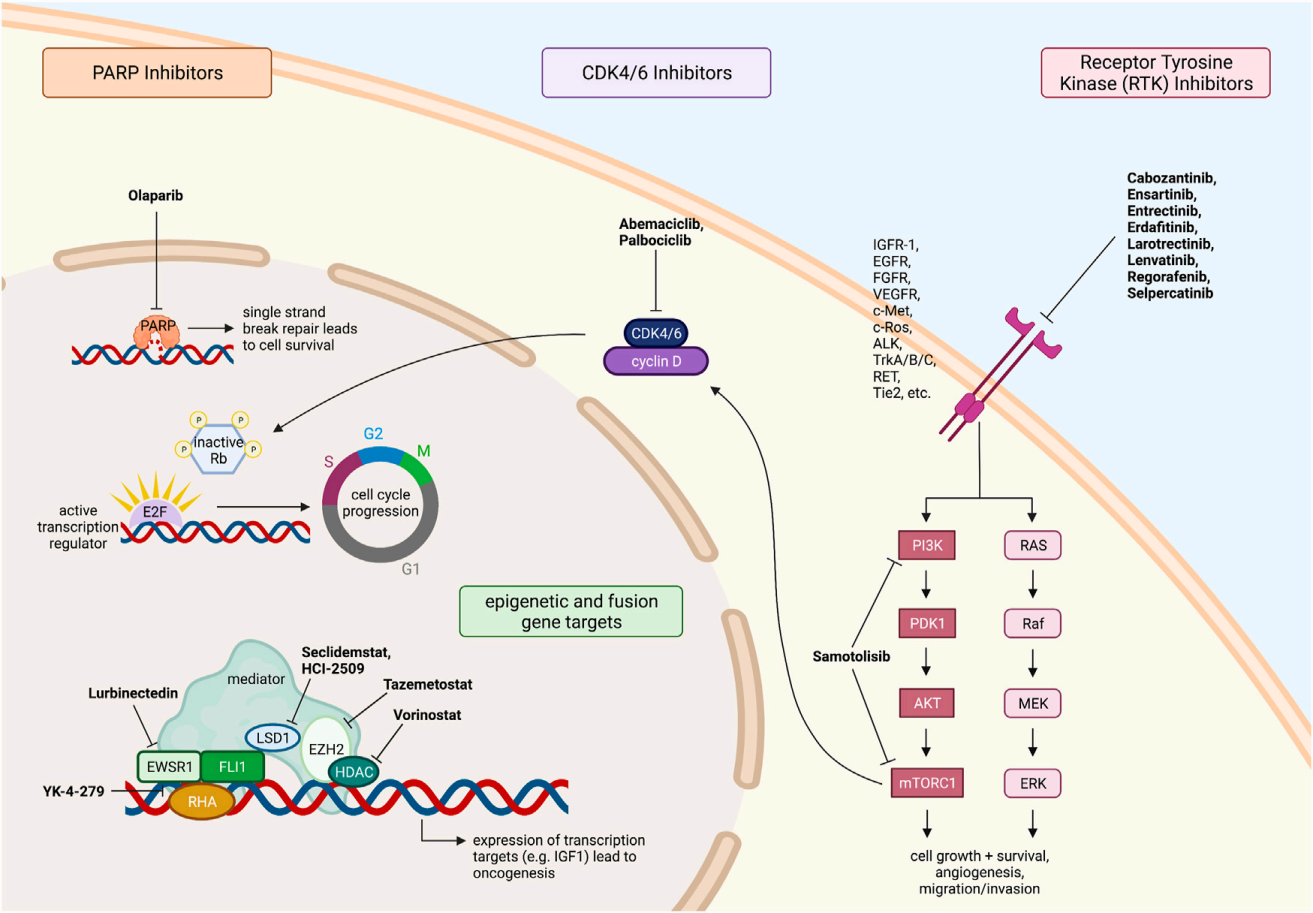
Fusion driven pediatric solid tumors with clinically actionable targets. Ewing sarcoma therapeutic targets and drugs under investigation. All figures were created with BioRender.com.

Another therapy, recently approved by the FDA in 2020, is lurbinectedin. Lurbinectedin is an inhibitor of RNA polymerase II and induces DNA breaks in cells that result in apoptosis (Markham, 2020). This small molecule therapy covalently binds to guanine, located centrally in the minor groove of DNA, forming adducts capable of inducing DNA double-strand breaks (Markham, 2020). Lurbinectedin is also being investigated as to whether it may induce immunogenic cell death and increase anti-tumor immunity. This is potentially due to the fact that lurbinectedin has been associated with a reduction in tumor associated macrophages and monocytes in pre-clinical *in vitro* and *in vivo* models (Markham, 2020). Lurbinectedin has been shown to be an effective anti-tumor treatment in solid tumors, and it is well tolerated by patients. Currently there is one current trial testing lurbinectedin in combination with irinotecan against solid tumors in adults but not in the pediatric population (clinicaltrials.gov, NCT02611024). The trial is based on 2016 laboratory studies where lurbinectedin was shown to cause nuclear redistribution of the *EWSR1-FLI1* translocation, resulting in less activity at the promoter and lower levels of mRNA and protein (Harlow et al., 2016). This effect was confirmed in xenograft studies, and when lurbinectedin was combined with irinotecan, there was a complete reversal of EWSR1-FLI1 tumorigenic activity, as ES cells were replaced with benign fat cells. The elimination of tumors in 30–70% of mice in only 11 days from the inception of treatment was observed (Harlow et al., 2016). The targeted therapies discussed above for ES are summarized in Figure 1.

### Rhabdomyosarcoma

Rhabdomyosarcoma (RMS), derived from primitive mesenchymal cells in the striated skeletal muscle lineage and myogenic progenitors, is the most common soft tissue sarcoma in children.

For localized RMS cases, gross surgical resection of the primary tumor plus radiation therapy and chemotherapy is considered standard-of-care, although long-term toxicities from RT is a concern in younger patients. Over 90% of low-risk localized RMS patients have relapse-free survival with cytotoxic multi-agent chemotherapy. Most recently vinorelbine and low-dose cyclophosphamide maintenance therapy was trialed in high-risk localized RMS patients and demonstrated improvement in overall survival (Chen et al., 2019). However, overall survival for metastatic and recurrent RMS remains low at 21 and 30%, respectively, indicating the need for novel targeted therapies to improve survival outcomes (Chen et al., 2019).

Alveolar RMS (ARMS), harboring an oncofusion, is the most aggressive subtype due to its high metastasis and recurrence rates (van Erp et al., 2018). ARMS tumors are characterized by the presence of a chromosomal translocation, either the *PAX3-FOXO1* fusion gene or the *PAX7-FOXO1* fusion gene. The *PAX3-FOXO1* rearrangement fuses the DNA-binding domain of paired box gene 3 (*PAX3*) on chromosome 2 to the transactivation domain of forkhead box protein O1 (*FOXO1*) on chromosome 13 (van Erp et al., 2018). The PAX-FOXO1 fusion protein is an aberrant oncogenic transcription factor, resulting in downstream transcription and translation of proteins involved in oncogenic transformation (Chen et al., 2019).

The PAX-FOXO1 transcription factor, which is both upstream to many of the signal cascades that promote RMS tumorigenesis, is a direct and promising target, although inhibitors that bind to PAX-FOXO1 with good specificity and affinity have yet to be designed. Another way to suppress PAX-FOXO1 transcription factor activity is through epigenetic targets, with therapy aimed at inhibiting the co-regulators and chromatin-remodeling complexes involved in transcription. Small molecule inhibitors such as JQ1 that target the BET bromodomain-containing protein (BRD4), an epigenetic reader that mediates transcription, can disrupt BRD4 and PAX3-FOXO1 interaction, leading to degradation of PAX3-FOXO1 and reduced transcription of the oncogenic fusion protein (Gryder et al., 2017). Histone deacetylase (HDAC) inhibitors such as entinostat, panobinostat, and vorinostat have also been shown to delay tumor growth in xenograft RMS models (Hedrick et al., 2015).

Receptor tyrosine kinases (RTKs), such as IGFR1, VEGFR, EGFR, FGFR4, and PDGFR*α* become constitutively activated and cause downstream tumorigenic effects in the presence of PAX-FOXO1 gene fusions and have also been identified as potential targets in RMS. Recently completed and currently ongoing clinical trials that target these RTKS are summarized in Table 3.

**TABLE 3:** Small Molecule Clinical Trials that are Recruiting or Active for Pediatric and Adolescent Rhabdomyosarcoma Patients (All Data from ClinicalTrials.gov).

Name of study	Phase	Target(s)	Small molecule treatment	Identifier
9-ING-41 with Chemotherapy in Sarcoma	II	GSK-3β	9-ING-41	NCT05116800
Ensartinib in treating patients with Relapsed or Refractory Advanced Solid Tumors, Non-Hodgkin Lymphoma, or Histiocytic disorders with ALK or ROS1 Genomic alterations (A Pediatric MATCH Treatment Trial)	II	ALK	Ensartinib	NCT03213652
Targeted Therapy Directed by Genetic testing in Treating Pediatric patients with Relapsed or Refractory Advanced Solid Tumors, Non-Hodgkin Lymphomas, or Histiocytic disorders (The Pediatric MATCH Screening Trial)	II	FGFR, ALK, IDH1, NTRK, PARP, CDK4/6, PI3K/mTOR, RET, MEK, EZH2, HRAS, MAPK, BRAF	Ensartinib, erdafitinib, ivosidenib, larotrectinib, olaparib, palbociclib, samotolisib, selpercatinib, selumetinib, tazemetostat, tipifarnib, ulixertinib, vemurafenib	NCT03155620
Erdafitinib in Treating Patients with Relapsed or Refractory Advanced Solid Tumors, Non-Hodgkin Lymphoma, or Histiocytic Disorders with FGFR Mutations (A pediatric MATCH Treatment Trial)	II	FGFR	Erdafitinib	NCT03210714
Ivosidenib in treating patients with Advanced Solid Tumors, Lymphoma, or Histiocytic Disorders with IDH1 Mutations (A Pediatric MATCH Treatment Trial)	II	IDH1	Ivosidenib	NCT04195555
Larotrectinib in treating patients with Relapsed or Refractory Advanced Solid Tumors, Non-Hodgkin Lymphoma, or Histiocytic disorders with NTRK Fusions (A Pediatric MATCH Treatment Trial)	II	NTRK	Larotrectinib	NCT03213704
Olaparib in Treating Patients with Relapsed or Refractory Advanced Solid Tumors, Non-Hodgkin Lymphoma, or Histiocytic Disorders with Defects in DNA Damage Repair Genes (A Pediatric MATCH Treatment Trial)	II	PARP	Olaparib	NCT03233204
Palbociclib in Treating Patients with Relapsed or Refractory Rb Positive Advanced Solid Tumors, Non-Hodgkin Lymphoma, or Histiocytic Disorders with Activating Alterations in Cell Cycle Genes (A Pediatric MATCH Treatment Trial)	II	CDK4/6	Palbociclib	NCT03526250
SARC024: A Blanket Protocol to study oral Regorafenib in patients with selected Sarcoma subtypes	II	VEGFR, PDGFR-β, FGFR, KIT, RET, RAF	Regorafenib	NCT02048371
Samotolisib in treating patients with Relapsed or Refractory Advanced Solid Tumors, Non-Hodgkin Lymphoma, or Histiocytic disorders with TSC or PI3K/MTOR Mutations (A Pediatric MATCH Treatment Trial)	II	PI3K, mTOR	Samotolisib	NCT03213678
Selpercatinib for the Treatment of Advanced Solid Tumors, Lymphomas, or Histiocytic disorders with activating RET Gene alterations, a Pediatric MATCH Treatment Trial	II	RET	Selpercatinib	NCT04320888
Sirolimus in combination with Metronomic Chemotherapy in children with recurrent and/or refractory Solid and CNS Tumors (AflacST1502)	II	mTOR	Sirolimus	NCT02574728
Tipifarnib for the Treatment of Advanced Solid Tumors, Lymphoma, or Histiocytic Disorders with HRAS Gene alterations, a Pediatric MATCH Treatment Trial	II	HRAS	Tipifarnib	NCT04284774
Vemurafenib in treating patients with Relapsed or Refractory Advanced Solid Tumors, Non-Hodgkin Lymphoma, or Histiocytic disorders with BRAF V600 Mutations (A Pediatric MATCH Treatment Trial)	II	BRAF	Vemurafenib	NCT03220035
Insulin-like Growth Factor 1 Receptor (IGF-1R) Antibody AMG479 (Ganitumab) in combination with the Src family kinase (SFK) inhibitor dasatinib in people with Embryonal and Alveolar Rhabdomyosarcoma	I/II	IGF1R, SFK	Ganitumab, dasatinib	NCT03041701
Vincristine and Temozolomide in combination with PEN-866 for Adolescents and young adults with Relapsed or Refractory Solid Tumors	I/II	HSP90	PEN-866	NCT04890093
Prexasertib, Irinotecan, and Temozolomide in people with Desmoplastic small round cell Tumor and Rhabdomyosarcoma	I/II	CHK1, CHK2	Prexasertib	NCT04095221
Onivyde with Talazoparib or Temozolomide in children with recurrent Solid Tumors and Ewing Sarcoma	I/II	PARP	Talazoparib	NCT04901702
A study of Abemaciclib in combination with Temozolomide and Irinotecan and Abemaciclib in combination with Temozolomide in children and young adult participants with Solid Tumors	I	CDK4/6	Abemaciclib	NCT04238819
Dose Escalation Study of CLR 131 in children, Adolescents, and young adults with Relapsed or Refractory Malignant Tumors Including but not limited to Neuroblastoma, Rhabdomyosarcoma, Ewing Sarcoma, and Osteosarcoma (CLOVER-2)	I	Lipid rafts	CLR 131 (phospholipid drug conjugate)	NCT03478462
Mocetinostat with Vinorelbine in Children, Adolescents and young adults with Refractory and/or Recurrent Rhabdomyosarcoma	I	HDAC	Mocetinostat	NCT04299113
Phase I study of Olaparib and Temozolomide for Ewing Sarcoma or Rhabdomyosarcoma	I	PARP	Olaparib	NCT01858168
Study of Palbociclib Combined with Chemotherapy in Pediatric Patients with Recurrent/Refractory Solid Tumors	I	CDK4/6	Palbociclib	NCT03709680
Vorinostat in combination with Chemotherapy in Relapsed/Refractory Solid Tumors and CNS Malignancies (NYMC195)	I	HDAC	Vorinostat	NCT04308330

A phase II clinical trial compared the IGF-1R inhibitor cixutumumab to conventional multi-agent chemotherapy in pediatric and adult patients with metastatic alveolar and embryonal RMS. Event-free survival (EFS) at 18 months was initially higher in patients receiving cixutumumab treatment (68%) compared to combinational chemotherapy (39%), but at 3-year follow-up, cixutumumab therapy had an event-free survival rate of 16% (Malempati et al., 2015; van Erp et al., 2018; Malempati et al., 2019). A phase II trial of the IGF-1R inhibitor ganitumab in combination with the Src Family Kinase (SFK) inhibitor dasatinib is ongoing in patients with relapsed or refractory RMS (clinicaltrials.gov, NCT03041701).

In a phase II clinical trial comparing conventional chemotherapy against an experimental arm adding the VEGFR inhibitor bevacizumab to cytotoxic chemotherapy in pediatric and adolescent patients with metastatic RMS and soft-tissue sarcomas, median EFS was higher in patients treated with bevacizumab (20.6 months, compared to 14.9 months for chemotherapy only). Higher objective response with bevacizumab was also observed at 54%, compared to 36% in the chemotherapy cohort, but findings were not statistically significant (Chisholm et al., 2017). In another phase I clinical trial evaluating the efficacy of bevacizumab with sorafenib and low-dose cyclophosphamide, 1 of 2 patients with refractory/recurrent RMS experienced a partial response (Navid et al., 2013).

EGFR is over-expressed in about 15–20% of ARMS tumors. Although no *in vivo* experiments targeting EGFR have been conducted yet, *in vitro* experiments in ARMS cell lines found that combination therapy of the anti-EGFR antibody cetuximab and the standard chemotherapy dactinomycin had synergistic effects in inhibiting cell growth and inducing apoptosis, with greater anticancer toxicity than dactinomycin alone (Yamamoto et al., 2013).

PAX3-FOXO1 also amplifies *FGFR4* expression in ARMS, and *FGFR4* has been implicated in resistance to apoptosis in tumors treated with therapeutics targeting the IGF1R-PI3K-mTOR pathway. However, the FGFR4 inhibitor BGJ398 showed synergy with IGF-1R inhibitor AEW54 in an ARMS cell line, suggesting that FGFR4 may be a promising target in treatment resistance prevention (Wachtel et al., 2014). Relapsed ARMS has also been shown to be responsive to pazopanib, a multi-kinase inhibitor currently in use for soft tissue sarcomas (Heske and Mascarenhas, 2021).

In the Children’s Oncology Group phase II clinical trial ARST0921, treatment of first-relapse RMS patients with a vinorelbine and cyclophosphamide chemotherapy backbone in combination with either the mTOR inhibitor temsirolimus or the VEGFR inhibitor bevacizumab resulted in 6-month event-free survival rates of 69 and 55%, respectively, with a greater proportion of partial responses in patients treated with temsirolimus (Heske and Mascarenhas, 2021). This data was promising enough to lead to further investigation of temsirolimus in the upfront setting for patients with newly diagnosed intermediate-risk RMS. This randomized phase III trial compares conventional cytotoxic chemotherapy with VAC/VI (alternating vincristine, dactinomycin, cyclophosphamide, and vincristine, irinotecan), with the addition of temsirolimus to the conventional VAC/VI chemotherapy backbone (Heske and Mascarenhas, 2021).

Developmental pathways may also provide therapeutic targets in RMS. GLI transcription factor activation through the constitutively active Hedgehog signaling pathway in RMS is involved in tumorigenesis. ERMS and ARMS xenograft models treated with GLI1/2 inhibitor GANT-61 combined with either temsirolimus or vincristine showed inhibition of proliferation via cell cycle arrest at the G0/G1 phase and significant reductions in tumor growth (Srivastava et al., 2014). Further research is needed to establish the potential of GLI inhibitors in RMS treatment.

Therapeutic targets may also lie in the apoptosis pathway. The Bcl-2 family of apoptotic proteins is involved in cancer cell survival and proliferation, and combination of Bcl-2 inhibitor ABT-737 and mTOR inhibitor AZD8055 was highly synergistic in inducing caspase-dependent apoptosis in ARMS and ERMS cells (Preuss et al., 2013).

**FIGURE 2:**
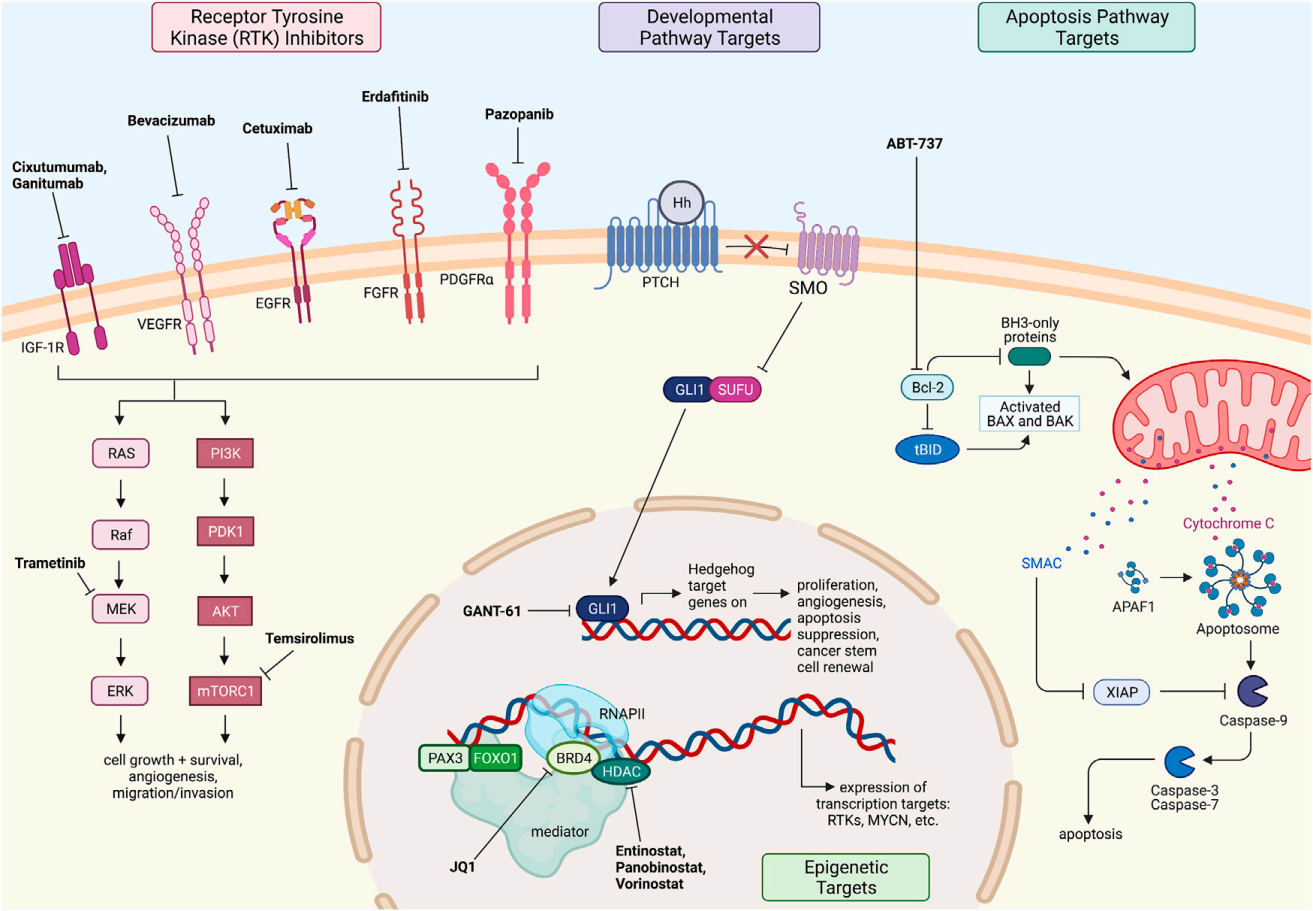
Fusion driven pediatric solid tumors with clinically actionable targets. Rhabdomyosarcoma therapeutic targets and drugs under investigation. All figures were created with BioRender.com.

Numerous targets for RMS are being investigated, yet there are currently no clinically impactful novel agents for the treatment of RMS. Inhibitors of the PAX-FOXO1 fusion protein have yet to be developed as well, despite the discovery of this oncogenic driver since 1993 (Galili et al., 1993). As prognosis for recurrent and metastatic RMS remains poor, this is an area of research that greatly needs further investigation and analysis of both *in vivo* and *in vitro* studies and clinical trials. The targeted therapies discussed above for RMS are summarized in Figure 2.

### *NTRK*-Fused Infantile Fibrosarcoma

Infantile fibrosarcoma (IFS) is a soft tissue sarcoma that presents as a localized, but large and rapidly growing tumor in the neonatal setting. IFS accounts for 24.5% of all soft tissue sarcomas found in children under the age of one. IFS has a very good overall prognosis, with studies demonstrating a 5-year survival rate of approximately 90%.

The optimal form of treatment for IFS is surgical resection, but wide local excision often results in mutilating surgery. In particular cases, neoadjuvant chemotherapy may be used to improve the probability of achieving a complete surgical resection; radiotherapy is used when surgical resection is deemed impossible (Parida et al., 2013). Thus there is a need for novel therapies in those IFS patients with extensive or unresectable disease, as well as those with metastatic IFS.

Nearly all IFS tumors harbor a neurotrophic tyrosine receptor kinase (*NTRK*) fusion, with 70% of IFS cases containing the ETS Variant Transcription Factor 6 (*ETV6*)-*NTRK3* gene rearrangement (Knezevich et al., 1998). In *NTRK* rearrangements, the 5’ fusion partner induces ligand-independent constitutive activation of the tropomyosin receptor kinase (TRK). This leads to uninterrupted downstream signaling of the RAS/RAF/MEK/ERK and PI3K/AKT pathways, which promote cancer cell survival, invasion, and proliferation (Amatu et al., 2019).

Larotrectinib is a TRKA, TRKB, and TRKC inhibitor that prevents neurotrophin-TRK interaction and activation, inducing apoptosis and inhibition of tumor growth. Larotrectinib has been approved by the FDA for use in children and adults with *NTRK*-fusion-positive tumors that are metastatic and/or will result in severe morbidity upon surgical removal and do not have any resistance mutations (Dunn, 2020).

Various clinical trials have shown efficacy of larotrectinib against *ETV6-NTRK3* fusion-positive IFS with negligible toxicity. In a phase I clinical trial, a 16-month-old female patient with refractory IFS was treated with larotrectinib. One month later, MRI scans revealed a reduction in tumor size by over 90%, and continued clinical response was also observed in later cycles of therapy (Nagasubramanian et al., 2016). Multiple phase 1 studies and case reports show reduction in tumor size, facilitating complete surgical resection, and remarkable reduction in size in other cases without surgical intervention (Caldwell et al., 2020).

Larotrectinib can also be used as a pre-surgical therapy to reduce tumor size, preventing radical and disfiguring surgeries. Larotrectinib treatment resulted in rapid and durable tumor regression and allowed for limb-sparing surgeries in patients who otherwise would have undergone amputations, confirming larotrectinib as a promising therapy for *ETV6-NTRK3* fusion-positive IFS (DuBois et al., 2018). In a multi-center, phase I clinical study enrolling eight NTRK-fusion-positive IFS patients between 1 month and 21 years of age, larotrectinib was well-tolerated and demonstrated antitumor activity in all eight patients. Four patients avoided disfiguring surgery and instead underwent R0 (negative resection margins with no tumor at marked resection region) and R1 (microscopic residual tumor at resection margin) surgical resection following larotrectinib treatment, indicating that larotrectinib may represent a new standard of care in controlling disease without morbid surgery (Laetsch et al., 2018).

**FIGURE 3:**
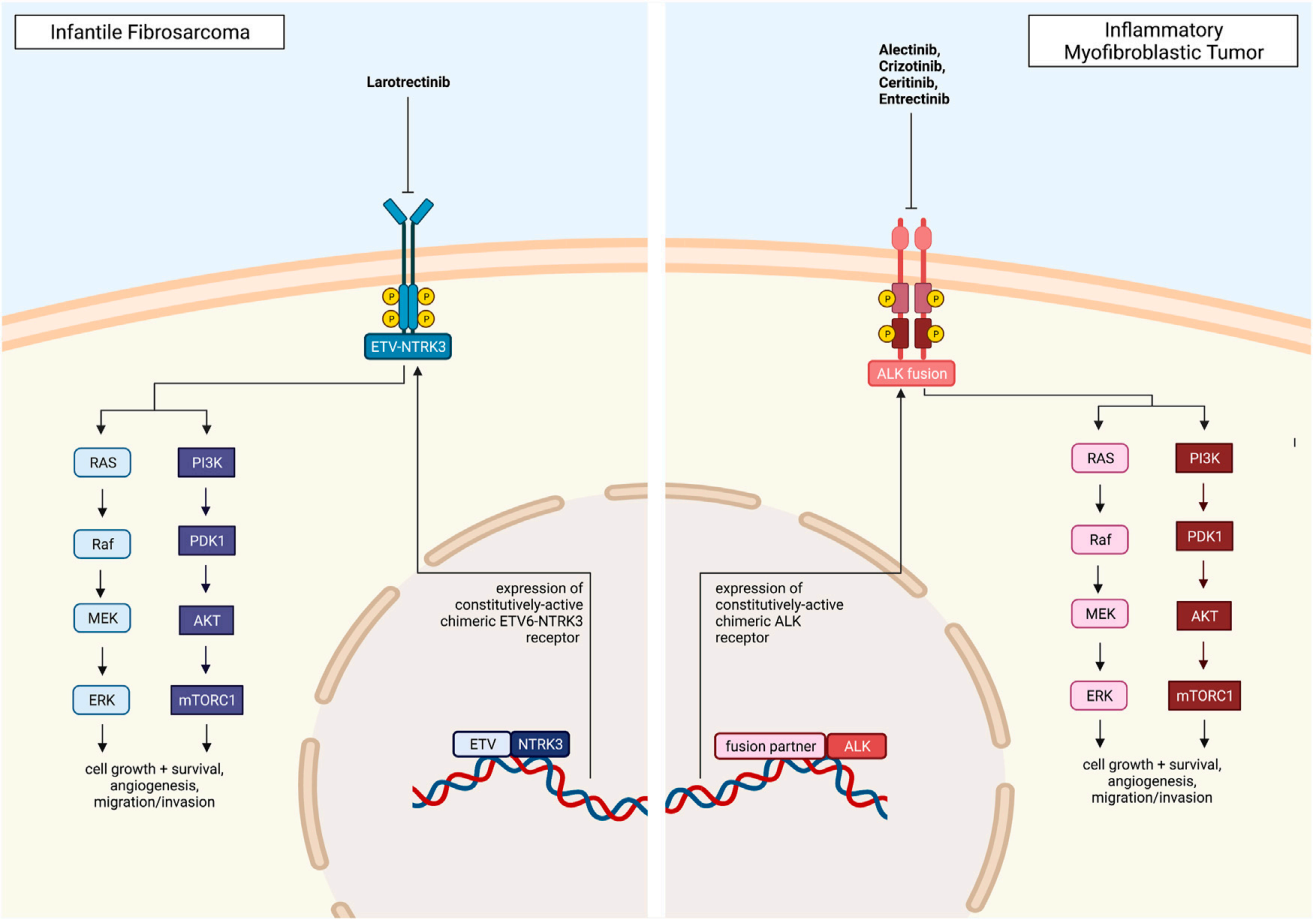
Pediatric solid tumors with a direct, targetable fusion kinase, including Infantile Fibrosarcoma and Inflammatory Myofibroblastic Tumor, and clinically approved drugs. All figures were created with BioRender.com.

Another gene fusion identified in IFS is the non-classical *LMNA-NTRK1* fusion. Crizotinib, an ALK inhibitor, has been shown to be effective in treating these tumors. Case reports show patients with refractory and metastatic IFS on crizotinib therapy had responses ranging from stable disease to complete and durable responses (Wong et al., 2016; Bender et al., 2019). The targeted therapies discussed above for IFS are summarized in Figure 3.

### Alveolar Soft Part Sarcoma

Alveolar soft part sarcoma (ASPS) is a rare, slow-growing but highly-angiogenic soft tissue sarcoma that often remains undetected until metastasis. ASPS is characterized by the *TFE3-ASPSCR1* fusion gene, which induces overexpression of the MET receptor tyrosine kinase. This leads to downstream activation of MEK1/2 and AKT, which promote tumor cell proliferation and angiogenesis (Mitton and Federman, 2012).

The 5-year survival rates of localized and metastasized ASPS are 80% and 10–40%, respectively. The main form of treatment for ASPS is surgical removal; radiation therapy may also be used to prevent regrowth following surgery. For unresectable tumors, however, ASPS is resistant to conventional chemotherapies, indicating the need for novel therapeutic targets such as receptor tyrosine kinase inhibitors and other anti-angiogenic agents in the treatment of ASPS (Mitton and Federman, 2012).

Tivantinib (ARQ 197), a selective inhibitor of MET receptor tyrosine kinase, has been investigated in a Phase II clinical trial involving 27 patients ≥13 years with metastatic and/or surgically unresectable ASPS. After 10 cycles of tivantinib, 78% of ASPS patients had stable disease for at least 4 months, and median PFS was 6 months (Wagner et al., 2012).

Sunitinib, a PDGFR and VEGFR tyrosine kinase inhibitor that reduces tumor angiogenesis and triggers cancer cell apoptosis, has demonstrated efficacy in controlling progressive metastatic ASPS in adults. In a case series of 10 adult patients, with a median age of 24, treated with daily sunitinib, treatment was well-tolerated and induced long-lasting responses. Partial response and stable disease were confirmed in 9 cases after 6 months, and median PFS was 17 months (Stacchiotti et al., 2011). A further case study reporting a patient with refractory metastatic ASPS treated with sunitinib described complete primary tumor regression and stabilization of the metastases (Ghose et al., 2012). These anecdotal reports of sunitinib efficacy in adult ASPS tumors suggest a potential therapeutic role of sunitinib in pediatric ASPS patients as well.

Cediranib, a potent inhibitor of VEGFR tyrosine kinase, has also been evaluated in treating adult ASPS. In a phase II clinical trial involving 46 patients, with a median age of 27, with unresectable and metastatic ASPS, 35% had partial response and 60% had stable disease after 24 weeks of daily cediranib treatment (Kummar et al., 2013).

### Inflammatory Myofibroblastic Tumor

Inflammatory myofibroblastic tumors (IMT) are rare soft tissue neoplasms made up of myofibroblastic spindle cells, typically characterized by benign local recurrence and rare metastasis. The standard treatment for IMT is complete surgical resection, which has favorable prognosis with most patients surviving past 10 years after complete tumor removal. However, therapy remains lacking for malignant unresectable IMT, especially since chemotherapy and radiation are not particularly effective (Maruyama et al., 2017).

About half of all IMTs are anaplastic lymphoma kinase (ALK) fusion-positive, consisting of cells that express an *ALK* gene rearranged with over 30 different identified 5’ fusion partners (Coffin et al., 2001). Rearrangement of *ALK* over-activates ALK receptor tyrosine kinase, which leads to downstream activation of the PI3K/AKT, JAK/STAT, and Ras/ERK pathways, increasing cancer cell proliferation and survival.

Crizotinib, a receptor tyrosine kinase inhibitor, inhibits ALK phosphorylation and its downstream signaling pathways, leading to G1/S cell cycle arrest and apoptosis. Crizotinib is currently used to treat *ALK*-rearranged cancers, such as anaplastic large-cell lymphoma (ALCL) and non-small cell lung cancer (NSCLC), and crizotinib has demonstrated efficacy in treating ALK-positive IMT as well.

In a 2017 study involving 14 unresectable, ALK-positive IMT pediatric patients who were given two doses of oral crizotinib daily, complete and partial responses were observed in five and seven patients, respectively (Mossé et al., 2017). In a cohort of eight ALK-positive IMT patients diagnosed with IMT between 2009-2016 and treated with crizotinib, four achieved a complete response, 3 a partial response, and one patient had stable disease. When preoperative crizotinib was used to decrease tumor size prior to surgical resection, 2 of the 3 partial responses achieved complete response. Furthermore, seven of eight patients were still alive at long-term follow-up, with no evidence of disease in six patients. Crizotinib was also found to be well-tolerated. As such, crizotinib combined with surgical resection appears to be effective in long-term disease control of ALK-positive IMT (Trahair et al., 2019).

For patients with *RANBP2-ALK* fusion positive epithelioid inflammatory myofibroblastic sarcoma (eIMS), a malignant variant of *ALK*-fusion-positive IMT, CD30 and ALK combination therapy may have high therapeutic potency. *RANBP2-ALK* eIMS xenografts treated with brentuximab-vedotin, targeting CD30 ^+^ tumor cells, and crizotinib resulted in tumor shrinkage and prolonged disease-free survival. In the diagnosis eIMS model, the majority of mice were confirmed as tumor-free 180 days past the study end date. However, disease recurrence was observed in all mice in the relapsed eIMS model, indicating that CD30 and ALK combination therapy may be most effective as early treatment for eIMS (Fordham et al., 2020).

Ceritinib, another ALK receptor tyrosine kinase inhibitor, has demonstrated efficacy against crizotinib-resistant tumors, with stronger potency in both crizotinib-naïve and crizotinib-refractory patients. Ceritinib is currently approved for use in ALK-positive NSCLC, but it has also shown excellent disease control in various ALK-positive IMT case studies (Santarpia et al., 2017). Recent case reports also show response in stage IV IMT or unresectable IMTs with ceritinib therapy and tumor response, allowing for surgical resection or complete response with ceritinib therapy alone (Mittal et al., 2021).

Alectinib is another ALK-inhibitor that has demonstrated efficacy against ALK-fusion-positive IMT. A case study in which a 26-year-old male with hyper-progressive ALK-fusion-positive IMT experienced a significant and durable response to treatment with alectinib was reported in 2017 (Saiki et al., 2017).

Entrectinib is an inhibitor of TRK, ROS1, and ALK, and has been shown to induce rapid and durable anti-cancer responses in NTRK, ROS1, and ALK-fusion tumors. A 16-year-old female patient with unresectable DCTN1-ALK-fusion-positive IMT had a complete response to entrectinib therapy, which controlled disease with low toxicity (Ambati et al., 2018).

Other gene translocations observed in IMT include proto-oncogene tyrosine-protein kinase (*ROS1*) fusions, which lead to tumorigenesis in mechanisms similar to ALK fusions. In a case study reported in 2021, the ALK- and ROS1-inhibitor lorlatinib was reported successful in treating a patient with refractory *TFG-ROS1* fusion-positive IMT (Carcamo et al., 2021).

### Fibrolamellar Hepatocellular Carcinoma

Fibrolamellar hepatocellular carcinoma (FL-HCC) is a rare form of hepatocellular carcinoma (HCC), affecting adolescents and young adults with no prior history of liver disease or risk factors for live cancer. In FL-HCC, a ∼400 kilobase deletion on chromosome 19 gives rise to the DnaJ Heat Shock Protein Family (HSP40) Member B1 (DNAJB1)-Protein Kinase CAMP-Activated Catalytic Subunit Alpha (PRKACA) fusion gene, and the corresponding *DNAJB1-PRKACA*-fusion protein has upregulated protein kinase activity to promote tumorigenesis (Honeyman et al., 2014).

Current treatments for FL-HCC remain in development. Chemotherapy for unresectable hepatocellular carcinoma (HCC) is a combination of atezolizumab and bevacizumab, but this therapy showed no clinical benefits in case studies in two patients with advanced FL-HCC (Al Zahrani and Alfakeeh, 2021). Sorafenib is another multi-tyrosine kinase inhibitor in use against advanced HCC; however, sorafenib has limited efficacy against FL-HCC, with only delayed progression of disease as the best response (Ang et al., 2013).

The only potentially curative treatment for FL-HCC is liver resection or liver transplantation; if the tumor is not completely removed, likelihood of recurrence is high. For patients with metastatic and/or unresectable disease, there are no effective treatments available and FL-HCC is progressive and fatal; median survival time is less than 12 months (Kassahun, 2016).

However, novel combination and targeted therapies are being investigated in the treatment of FL-HCC. In a phase II trial of continuous IV fluorouracil and thrice weekly recombinant interferon alfa-2b administered to eight FL-HCC patients, one patient had complete response while four had partial responses, and overall median survival was 23.1 months (Patt et al., 2003). In a case study involving a 27-year-old woman with FL-HCC, 10 cycles of gemcitabine and oxaliplatin were administered, and 5 years after the regimen had been completed, the woman remained in complete remission (Gras et al., 2012).

The PRKACA kinase domain was also found to play an essential role in FL-HCC tumor formation, suggesting the potential for small molecule PRKACA-inhibitors as a therapy for FL-HCC (Kastenhuber et al., 2017). However, further research is needed to investigate the efficacy and viability of PRKACA inhibitors against FL-HCC.

### Renal Cell Carcinoma

Pediatric translocation renal cell carcinoma (tRCC) is a rare kidney cancer found in children, accounting for 2–5% of all childhood renal neoplasms. Less-advanced tRCC tumors can be treated via surgical resection, whereas more-advanced tRCC is treated with targeted therapies.

The most common mutation associated with RCC is the *TFE3*-fusion gene, where the *TFE3* gene on chromosome Xp11.12 is rearranged with a fusion partner (i.e., PRCC, ASPSCR1, NONO, CLTC, SFPQ, etc.) from a different chromosome. The *TFE3*-fusion gene is a constitutively active promoter, causing dysregulated transcriptional TFE3 activity that leads to tumorigenesis through downstream upregulation of the PI3K/AKT/mTOR signaling pathway (Damayanti et al., 2018).

Cabozantinib is a small-molecule VEGFR2 and MET tyrosine kinase inhibitor that has already been approved for use in medullary thyroid cancer and renal cell carcinoma. For tRCC tumors that express MET, cabozantinib has been shown to regress disease with tolerable toxicity. In a 16-year-old female patient presenting with refractory, locally recurrent *TFE3*-fusion-positive tRCC with lung metastases, treatment with cabozantinib led to prompt and durable disease control. The patient was also noted to have reduction in pain, weight gain of 90% of weight previously lost on chemotherapy, and improved quality of life. In a 12-year-old male patient with stage IV *TFE3*-fusion-positive tRCC, treatment with cabozantinib demonstrated excellent disease control, including reduced pain just after 3 months and halted disease progression even 18 months later (Wedekind et al., 2017).

VEGFR tyrosine kinase and mTOR inhibitors, which are used in metastatic RCC therapies, have also demonstrated efficacy in disease control of tRCC. In a case study of 53 patients with tRCC, 21 patients who were *TFE3*-fusion-positive received targeted therapy. Fourteen patients treated with sunitinib had a median PFS of 8.2 months, with seven patients achieving either a partial or complete response. Eight patients treated with sorafenib had a median PFS of more than 6 months. Two pediatric patients had partial responses with single agent therapy of either sunitinib or sorafenib, and both patients continued with stable disease after 29 months. For patients that experienced disease progression while being treated with VEGFR inhibitors, switching to mTOR inhibitors temsirolimus and everolimus resulted in stable disease (Malouf et al., 2010).

### Synovial Sarcoma

Synovial sarcoma (SS) is a rare soft tissue sarcoma that occurs in adolescents and young adults, with a pathognomonic t (X; 18) (p11.2; q11.2) chromosomal translocation, fusing the *SS18* (formerly known as *SYT*) gene with the *SSX1*, *SSX2*, or *SSX4* gene (Clark et al., 1994; Shipley et al., 1994). Therapeutic approaches involve systemic chemotherapy with doxorubicin and ifosfamide combined with radiation therapy and/or surgery for local control.

The SS18-SSX fusion protein acts as an epigenetic modifier, driving tumorigenesis in SS cells (Kadoch and Crabtree, 2013). SWItch Sucrose Non-Fermentable (SWI/SNF) chromatin remodeling complexes, containing the BAF (BRG1 or BRM associated factors) complex, are disrupted in SS cells when the SS18-SSX fusion protein competitively replaces the wild-type SS18 in the BAF complex (Kadoch and Crabtree, 2013). The fusion SS18-SSX protein is found to co-localize with Polycomb Repressive Complexes 1 and 2 (PRC1, PRC2) (Soulez et al., 1999; Lubieniecka et al., 2008). PRC2 silences chromatin with its catalytic subunit, the histone methyltransferase Enhancer of Zeste 2 (EZH2) (Wilson et al., 2010). The relocalization of oncogenic BAF complexes to PRC repressed domains, recruitment of RNA Polymerase II, and initiation of transcription leads to epigenetic reprogramming and drives tumorigenesis (McBride et al., 2018).

Preclinical studies using EZH2 inhibitors in SS cell lines were promising, showing a decrease in cellular proliferation and migration *in vitro*, and with decreased tumor burden in xenograft models (Kawano et al., 2016; Shen et al., 2016). However, in a phase II clinical trial in adult patients with synovial sarcoma, the selective EZH2 inhibitor tazemetostat exhibited less promising preliminary results. Tazemetostat was well tolerated by patients with few adverse events, but the best response observed was stable disease, which occurred in 33% of patients (Schoffski P et al., 2017).

### Other Novel Approaches Such as Immunotherapy and ONC201/TIC10

Various strategies have been employed to promote the immune response against pediatric solid tumors. These include oncolytic virus-based therapy, antibody-dependent cellular cytotoxicity (ADCC), bispecific antibodies, immune checkpoint inhibitors, tumor microenvironment targeted therapies (cancer-associated fibroblasts, macrophages), cytokines, growth factors and CAR-T cells (Marayati et al., 2019).

TRAIL-Inducing Compound #10 (TIC10)/ONC201 is a novel agent that activates a potent innate immune pro-apoptotic anti-cancer response through the integrated stress response (Allen et al., 2013; Kline et al., 2016; Wagner et al., 2018; Prabhu et al., 2020). ONC201 has potential for further development in pediatric solid tumors including in combination with epigenetic modulators (Chang et al., 2020; Chang et al., 2021; Honeyman et al., 2021; Zhang et al., 2021).

## Conclusion

Pediatric non-CNS solid tumors are a diverse group of tumors that are best managed with a multi-disciplinary approach. Combinations of chemotherapy, surgery and radiation have improved patient outcomes (Ward et al., 2019b), but these approaches have changed little in the previous 2 decades. In addition, patients with unresectable, metastatic, and recurrent disease typically face dismal outcomes with current multimodal therapy. With the ability to analyze the genomes of individual patients and identify molecular drivers specific to an individual cancer, opportunities for clinical trials, such as the Pediatric MATCH trial run by the Children’s Oncology group, are now available. Currently, there exists promising novel therapies in preclinical investigation as well as ongoing clinical trials. Investigation continues in these targeted therapies, their toxicities, and administration in the pediatric population. Novel therapies, including small molecule agents, targeted therapies, and other precision medicine-based treatments, can offer an opportunity to examine unique mechanisms in treating pediatric sarcomas and other solid tumors.

Preclinical strategies to target gene fusions include both the utilization of existing inhibitors as well as the development of novel drugs through rational design approaches. The development of novel therapies requires a significant development time and multidisciplinary effort that includes expertise in genomics, molecular biology, pharmacology, clinical trials, and clinical oncology. Using these resources more effectively will not only help drive the development of novel therapies, but also better inform individual patient treatment decisions. As our understanding of the molecular drivers of pediatric fusion-positive cancers increases, so do our abilities to tailor therapies toward better outcomes for this patient population.

## Data Availability

The original contributions presented in the study are included in the article/Supplementary Material, further inquiries can be directed to the corresponding authors.
